# Clinical Patterns, Survival, Comorbidities, and Treatment Regimens in 149 Patients With Pemphigus in Tuscany (Italy): A 12-Year Hospital-Based Study

**DOI:** 10.3389/fimmu.2022.895490

**Published:** 2022-07-08

**Authors:** Lavinia Quintarelli, Alessio Coi, Roberto Maglie, Alberto Corrà, Elena Biancamaria Mariotti, Cristina Aimo, Valentina Ruffo di Calabria, Alice Verdelli, Beatrice Bianchi, Elena Del Bianco, Emiliano Antiga, Marzia Caproni

**Affiliations:** ^1^ Rare Skin Diseases Unit, Section of Dermatology, Department of Health Sciences, Unità Sanitaria Locale (USL) Toscana Centro, European Reference Network-Skin Member, University of Florence, Florence, Italy; ^2^ Unit of Epidemiology of Rare Diseases and Congenital Anomalies, Institute of Clinical Physiology, National Research Council, Pisa, Italy; ^3^ Department of Health Sciences, Section of Dermatology, University of Florence, Florence, Italy

**Keywords:** pemphigus, epidemiology, rituximab, comorbidities, drugs

## Abstract

**Introduction:**

Pemphigus encompasses a group of muco-cutaneous autoimmune bullous diseases characterized by the loss of adhesion between keratinocytes. The disease is associated with increased morbidity and mortality.

**Materials and Methods:**

We characterized clinical patterns, survival, comorbidities, and drug prescriptions in patients with pemphigus referred to the Section of Dermatology of the University of Florence from January 2010 to December 2021.

**Results:**

A total of 149 patients were identified (female/male sex ratio = 2.0). Median age at diagnosis was 57.7 ± 17.2 years; 108 patients were diagnosed with pemphigus vulgaris (PV) (72.5%) and 35 (23.5%) with pemphigus foliaceus (PF). Paraneoplastic pemphigus (PNP) and IgA-pemphigus accounted for three patients each. The overall survival rate was 86.9%. Accordingly, 14 (9%) patients died during the study period. The average age at death was 77.8 ± 9.3. Age at diagnosis was a risk factor for death in patients with pemphigus. Average concentration of Dsg3-IgG and Dsg1-IgG was 85.6 ± 68.8 and 75.9 ± 68.4, respectively. The most serious comorbid diseases included cerebro- and cardiovascular accidents and malignancies. Regarding the treatment regimen, we found a substantially stable use of systemic steroids in the 2010–2018 period; the prevalence of use of mycophenolic acid increased, whereas that of azathioprine decreased. The use of rituximab showed the highest increase in the 2013–2018 period. Proton-pump inhibitors and antibiotics were the most frequently prescribed non-immunomodulating drugs.

**Conclusions:**

In this large series of the patients, patients with pemphigus showed a high incidence of serious comorbid diseases, highlighting the importance of a multidisciplinary approach for a proper management of the patients. Rituximab was the immunomodulating drug showing the highest increase in use over time, reflecting the growing evidence of its efficacy as a first-line treatment in pemphigus.

## Introduction

Pemphigus is a heterogeneous group of autoimmune bullous diseases, characterized by autoantibodies targeting intra-epidermal adhesion molecules, particularly desmosomal cadherins, such as desmoglein (Dsg) 3 and Dsg1 (1). Antibody-antigen binding interferes with homophilic interactions between desmogleins, leading to the loss of adhesion between keratinocytes, also referred to as acantholysis (2–6).

Pemphigus vulgaris (PV) and pemphigus foliaceus (PF) are the two major variants of pemphigus. Pemphigus variants other than PV and PF are less frequent. These include pemphigus vegetans, paraneoplastic pemphigus (PNP), pemphigus herpetiformis (PH), and IgA pemphigus (2, 7).

The epidemiological characteristics of pemphigus vary according to the clinical variant, geographical regions, and ethnicities (3, 4, 8).

PV is considered the most prevalent type of pemphigus, corresponding to 70% of all cases (3, 4).

In European countries, the average age at onset of PV varies from 50 to 60 years (9). Conversely, PV is extremely rare during childhood (10).

PV seems to be more prevalent in female than male patients, with a female/male sex ratio (F/M SR) ranging from 1.1 in Finland to 5.0 in the USA (2, 4).

PF is divided in two different subtypes: sporadic and endemic. Sporadic PF is the second most common type of pemphigus, representing about 20% of pemphigus cases (2, 4). The average age at onset is around 50 years, with no preference for sex or ethnicity (3). Endemic PF is a variant of PF with a high incidence rate in some rural areas of Brazil, Colombia, and Tunisia (11).

Atypical pemphigus variants, including PNP, IgA pemphigus, and PH, are far less common. PNP accounts for 3%–5% of pemphigus cases (2, 12). The exact incidence and prevalence of PNP are difficult to evaluate. PNP is almost always associated with an underlying malignancy, particularly hematological malignancies including non-Hodgkin’s lymphomas, chronic lymphocytic leukemia, and Castleman’s disease. Rarely, PNP can also arise in association with solid tumors (13). The average age at onset ranges between 45 and 70 years (14), although it can also occur in children and adolescents, especially when associated with Castleman’s disease (15). Regarding gender, different data were reported in the literature: a French study reported a predominance of the male sex (58.5% of cases), whereas an international multicenter study including Asian patients reported a female predominance (56.7%) (16, 17).

Treatment of pemphigus largely relies on immunosuppressive treatments. High-dose systemic corticosteroids are considered frontline therapies and are necessary to achieve rapid clinical improvement. A variety of immunosuppressive treatments, including dapsone, azathioprine, mycophenolate, and cyclophosphamide, serves as steroid-sparing agents, allowing progressive tapering of systemic steroids but are less useful for the treatment of active disease (2).

Rituximab (RTX), a monoclonal antibody targeting CD20, shows a remarkable clinical efficacy, longer clinical remission, and significant steroid sparing effects in patients with pemphigus and is now regarded as a first-line treatment in patients with moderate to severe disease (5, 18).

Despite a drastic reduction in pemphigus mortality since the advent of systemic corticosteroids and immunosuppressive treatments, pemphigus associated mortality appears to be 1.7–3.6 higher than that observed in the general population (19).

One reason explaining the higher mortality of patients with pemphigus is linked to treatment-related adverse effects, such as severe infections. Other reasons may be related to associated comorbidities, particularly cardiovascular diseases and malignancies (20, 21).

Among pemphigus variants, PNP seems to be associated with the highest mortality, which is related to either the associated malignancy or the severe clinical course, characterized by a lower responsiveness to immunosuppressive regimens and the increased risk of systemic complications, such as bronchiolitis obliterans (22).

The purpose of this study is to characterize clinical and epidemiological characteristics of patients with pemphigus who attended our dermatologic clinic over a period of 12 years.

## Materials and Methods

### Patients

We conducted a 12-year retrospective study including 149 patients diagnosed with pemphigus at the Rare Skin Diseases Unit of Azienda USL Toscana Centro, University of Florence from January 2010 to December 2021. All cases were included into the Registry of Rare Diseases of Tuscany.

### Inclusion and Exclusion Criteria

Eligible for the study were all patients at every age that meet the criteria for the diagnosis of pemphigus proposed by current guidelines (5, 6).

Briefly, the diagnosis and classification of pemphigus was based on the clinical presentation and histopathological and immunopathological criteria, including i) detection of IgG or IgA intercellular deposits at direct immunoflouorescence microscopy from a perilesional tissue sample, ii) detection of circulating antibodies binding the inter-keratinocyte surface at indirect immunofluorescence, and/or iii) detection of IgG against desmosomal proteins, e.g., Dsg3 or Dsg1, by enzyme-linked immunosorbent assay or immunoblotting. Assessments of circulating anti-Dsg1 and anti-Dsg3 autoantibodies were performed using commercial kits (MBL MESACUP-2 TEST, Naka-Ku Nagoya Aichi, Japan).

Patients whose diagnosis of pemphigus could not be confirmed by the abovementioned criteria or who were not living in Tuscany at the time of diagnosis were excluded from the analysis.

### Demographic and Clinical Characteristics of the Patients

Data regarding the clinical characteristics and phenotype of the disease (PV, PF, PNP, and other rare variables), the demographic characteristics (age and sex), and the autoantibody profile at diagnosis (anti-Dsg1 and Dsg3 IgG autoantibodies) and within 12 months after diagnosis were collected from all the patients with pemphigus identified from the registry.

### Comorbidities and Pharmaceutical Prescriptions

A subset of 78 cases (out of 149) endowed of the regional unique anonymous identification number was linked to the regional hospital discharge records and the drug prescription database.

Associated comorbidities were extrapolated from hospital admissions.

We focused on various comorbidities that have been associated with pemphigus according to the literature. Associated comorbidities were identified using the International Classification of Diseases, Ninth Revision, Clinical Modification.

The drug prescription database contains information on dispensed drugs reimbursed by the National Health Service. Only outpatient prescriptions were collected in the database.

The prevalence of use of the most common classes of prescribed drugs in pemphigus was calculated for each year of the 2011–2018 period, by dividing the number of pemphigus cases with at least one dispensing of each pharmaceutical class for the number of prevalent cases at the beginning of each year. Drugs prescribed during 2010 were excluded to avoid underestimation related to patients diagnosed in the last months of 2010 and started to be treated from 2011.The Anatomical Therapeutic Chemical (ATC) classification system was used to code drugs information. Two macro-areas of drugs have been identified:1) those used for the treatment of pemphigus and 2) those used for the management of pemphigus-associated comorbidities.

The first group included the following: prednisone, dapsone, cyclophosphamide, azathioprine, mycophenolate mofetil, RTX, medium- and high-potency topical corticosteroids and analgesic, particularly opioids.

The second group included the following: bisphosphonates, antimicrobial drugs, proton-pump inhibitors (PPIs), beta blockers, calcium channel blockers, angiotensin converting enzyme (ACE) inhibitors and sartans, statins, antidiabetics including insulin, and antithrombotic drugs.

### Statistical Analysis

Differences in demographic (age and sex) and in anti-Dsg1 and anti-Dsg3 antibodies at T0 (baseline) and T1 were evaluated overall and by pemphigus variants using Student’s *t*-test for continuous variables and Fisher’s exact test for categorical variables. For continuous variables, mean values with standard deviation (SD) were reported in the text.

Survival estimates were calculated by sex, age class (<40 years, 40–59 years, 60–74 years, and ≥ 75 years), and pemphigus variants (PV and PF) using the Kaplan–Meier method, with the log-rank test to assess statistically significant differences.

The effects of sex, age at diagnosis, pemphigus variant, and levels of anti-Dsg1 and anti-Dsg3 at T0 were estimated using Cox proportional hazards regression model and hazard ratios (HRs) with 95% confidence intervals (CI).

The data were analysed with Stata, version 16.

Two-sided p-value < 0.05 was considered statistically significant in all analyses of this study.

## Results

### Demographic and Clinical Characteristics

A total of 149 patients with a new diagnosis of pemphigus was identified between January 1, 2010, and December 31, 2021. The population included 50 male (33.6%) and 99 female (66.4%) patients with a F/M SR of 2.0.

Of these 149 patients, 108 had PV (72.5%), 35 had PF (23.5%), three had PNP (2%), and three had IgA pemphigus (2%).

Oral PV was detected in 53 out of 108 patients with PV (49.07%).

**Figure 1:**
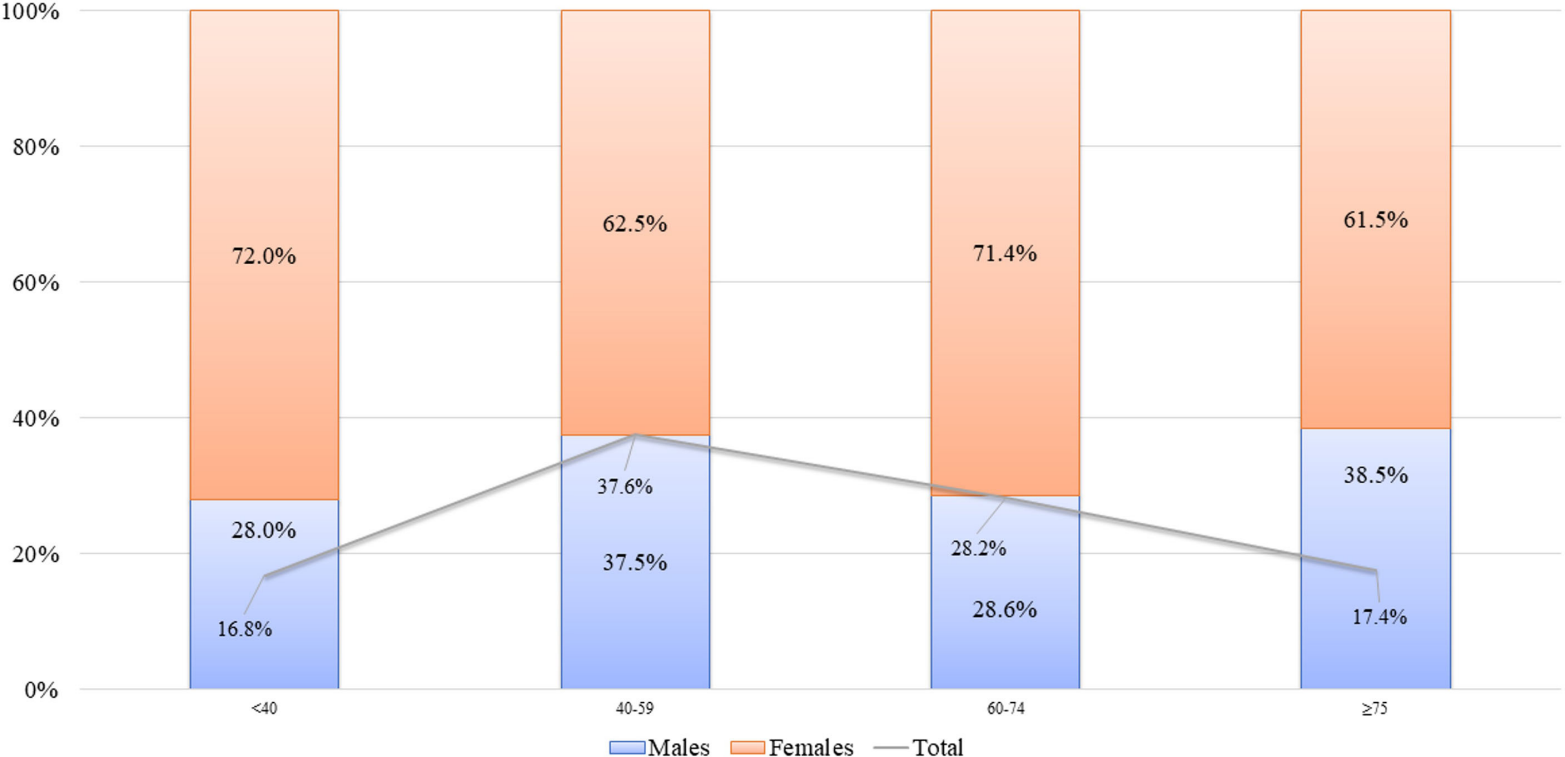
Age distribution of patients, total and by sex. Proportions of male and female patients for each age class are reported in bars. Total percentage of each age class is also reported (gray line).

In more details, among patients with PV, female patients (n = 69, 63.9%) were predominant over male patients (n = 39, 36.1%) with a F/M SR of 1.8. Among 35 patients with PF, 26 were female (74.3%) and nine were male (25.7%) (SR = 2.9). Among patients with PNP, there were two female (66.7%) and one male (33.3%) patients. Finally, there were two female (66.7%) and one male (33.3%) patients suffering from IgA pemphigus (33.3%). The mean age at diagnosis was 57.7 years ( ± 17.2, range: 6.3–89.9); in details, mean age at diagnosis was 59.0 ( ± 17.0) for male and 57.0 ( ± 17.4) for female patients, without significant differences (p = 0.51). Considering the clinical subtype, the mean age at diagnosis of PV was 56.5 ( ± 17.5) (male, 57.6 ± 18.3; female, 55.9 ± 18.3), whereas it was 59.9 ( ± 16.9) (male, 63.9 ± 11.0; female, 58.5 ± 18.5) for PF. At diagnosis, female patients were generally younger than male patients; however, the difference between both groups was not statistically significant (p-values >0.05) (**Figure 1**).

### Antibodies

The mean titer of circulating autoantibodies (reported as UI/ml) at the time of diagnosis (T0) was 75.9 ± 68.4 for anti-Dsg1 and 85.6 ± 68.8 for anti-Dsg3 antibodies. In the PV groups, anti-Dsg3 IgG antibodies were significantly higher in patients with mucocutaneous than oral PV (135.9 vs. 95.3, p = 0.0003), whereas the average value of anti-Dsg1 IgG antibodies was significantly lower in patients with oral PV than in patients with muco-cutaneous PV (8.6 vs. 111.6, p < 0.0001t). As expected, there were significant differences in the mean value of anti-Dsg1 at T0 between PV and PF (59.1 vs. 136.8, p < 0.0001) and in the mean value of anti-Dsg3 at T0 between PV and PF (115.0 vs. 13.0, p < 0.0001).

At T1, corresponding to the interval between diagnosis and the first 12 months of follow-up, a decrease in anti-Dsg1 antibodies was recorded in 71 out of 84 patients, and a decrease in anti-Dsg3 antibodies was recorded in 72 out of 83 patients (corresponding to 84.5% and 86.7% of the patients, respectively).

In particular, the average titers at T1 were 30.6 ± 46.8 for anti-Dsg1 and 51.7 ± 59.5 for anti-Dsg3 antibodies.

The mean value of decrease of anti-Dsg1 antibodies between T0 and T1 was −65.5 ± 60.0, with significant differences between PV and PF (−58.1 vs. −97.1, p = 0.02).

The mean value of decrease of anti-Dsg3 antibodies between T0 and T1 was −52.3 ± 48.8, also in this case with significant differences between PV and PF (−65.2 vs. −15.4, p < 0.001). We next evaluated whether immunosuppressive adjuvants induced different degree of autoantibody reduction after at least 270 days following treatment. Interestingly, we observed that patients receiving rituximab experienced a higher decrease of anti-Dgs1 antibodies (92.8 ± 70.5) than patients who did not received it (52.4 ± 68.4), with a difference approaching the statistical significance (p = 0.07). On the contrary, we did not observe statistically significant differences in the decrease of anti-Dsg3 antibodies between the two groups.

### Survival

During the study period, 14 (nine male and five female) out of 149 patients died (9.4%).

The average age at death was 77.8 ± 9.3 years (range: 56.2–94.8); 77.9 and 77.6 years for male and female patients, respectively. No deaths were observed for patients below 40 years.

The cause of death was retrieved in eight out of 14 patients. Five patients died due to complications related to an advanced cancer. One patient died due to an acute stroke, one died due to an acute cardiovascular event complicated by sepsis, and one died due to an *ab ingestis* pneumonitis.

**Figure 2:**
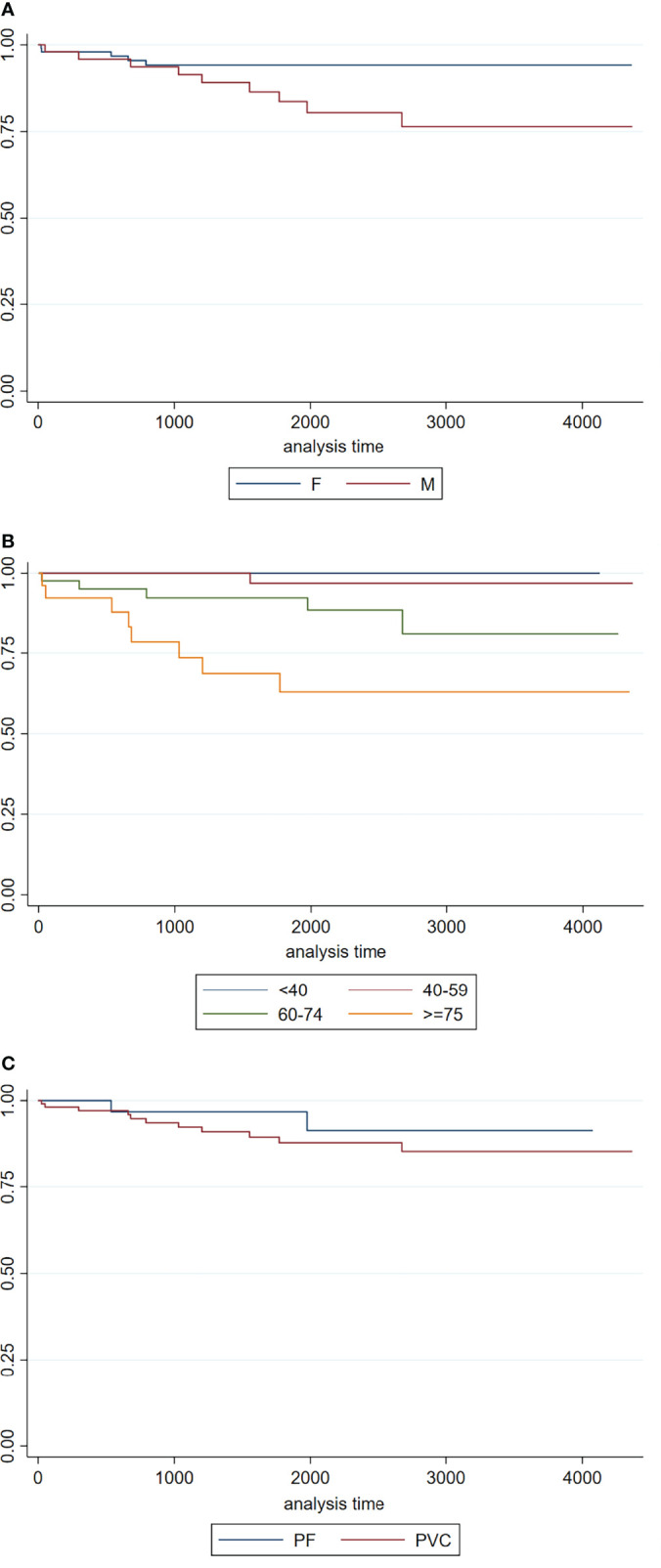
**(A)** Kaplan–Meier survival curves by sex (female and male patients in continue and dotted lines, respectively); **(B)** Kaplan–Meier survival curves by age classes (under 40, between 40 and 59, between 60 and 74, over 75); **(C)** Kaplan–Meier survival curves by pemphigus variants (PF and PV).

The Kaplan–Meier overall survival rate estimated during the study period was 86.9%. A significantly higher survival rate was observed in female than in male (94.2% vs. 76.4%, p = 0.03) (**Figure 2A**).

Survival rate significantly decreased from patients under 40 (100%) to patients between 40 and 59 years (96.9%), to patients between 60 and 74 years (81.0%), and to over 75 (62.9%) (p < 0.001) (**Figure 2B**).

Although survival rate was greater in the PF subtype than in PV (91.4% vs. 85.4%), the difference was not statistically significant (p = 0.39) (**Figure 2C**).

Cox proportional hazards regression showed that each additional year at diagnosis was associated with a 9% risk of dying (p < 0.001) and that male patients had a significantly increased risk of death than female patients (non-adjusted HR: 3.12; 95% CI: 1.04–9.33). However, after adjustment for age at diagnosis, the difference between male and female patients was not statistically significant.

The levels of anti-Dsg1 and anti-Dsg3 at T0 did not appear to be a risk factor for survival, even after adjustment for sex and age.

### Comorbidities

Cancer was found in nine out of 75 patients with pemphigus (12.0%); in detail, eight patients had received a diagnosis of a solid tumor (10.7%): among them, four cases (5.3%) occurred in patients prior to the diagnosis of pemphigus.

The associated solid malignancies included the following: eosophageus carcinoma (one patient); carcinoma of the hypopharynx (two patients); uterine leiomyoma (two patients); malignant neoplasm of the retroperitoneum and peritoneum in one patient; and bladder carcinoma (one patient).

A hematological malignancy was detected in one patient (1.3%).

Regarding autoimmune diseases, two cases of thyroiditis (2.7%) and four cases of arthritis (5.3%) were recorded, all in female patients and before the diagnosis of pemphigus.

Regarding neurological and psychiatric disorders, we found one patient who had been hospitalized due to a hereditary degenerative disorder of the central nervous system; one patient was hospitalized for encephalitis and two hospitalizations occurred for personality disorder. On the other hand, we have not found any cases of hospitalization for multiple sclerosis, epilepsy, organic psychotic conditions, such as dementia and alcohol- or drug-related mental disorders.

Regarding cerebro- and cardiovascular diseases, we found six (8.0%) hospitalizations for heart attack of which four (5.3%) occurred following the diagnosis of pemphigus; nine (12.0%) hospitalizations for non-ischemic heart disease, although none following the diagnosis of pemphigus; one hospitalization for pulmonary hypertension preceding the diagnosis of pemphigus was reported; finally, five (6.7%) hospitalizations for cerebral stroke of which one occurred in the period following the diagnosis of pemphigus was recorded.

About the vascular system, three (4.0%) hospitalizations for arterial vascular system disorders, two of these after the diagnosis of pemphigus, were detected; whereas five (6.7%) for venous and/or lymphatic system disorders, of which four after pemphigus diagnosis, were recorded.

Regarding the respiratory system, one case of acute infection of the upper respiratory tract and two hospitalizations for chronic obstructive pulmonary disease (COPD) were detected, both prior to the diagnosis of pemphigus; instead, two (2.7%) hospitalizations for pneumonia occurred following the diagnosis of pemphigus. None of them was taking either topical or systemic steroids at the time of hospitalization. No hospitalizations for pleural diseases were registered.

Eleven diagnosis of diabetes mellitus were recorded (14.7%; five male and six female patients) of which eight (10.7%) occurred after the diagnosis of pemphigus.

Regarding other metabolic disorders overweight and obesity were found in a total of eight (10.7%) patients, all registered prior to the diagnosis of pemphigus.

Regarding ocular diseases, three patients with glaucoma were registered, of one after the diagnosis of pemphigus (4.0% and 1.3% respectively); one patient was hospitalized due to cataracts (1.3%) diagnosed after the diagnosis of pemphigus.

A total of 11 patients (14.7%) diagnosed with genitourinary tract diseases were registered, of which three cases of nephritis registered after the diagnosis of pemphigus.

Regarding the gastrointestinal (GI) system, three patients suffered from an inflammatory disease of the upper GI tract (including esophagitis, gastritis, peptic ulcers, and duodenitis) occurred following the diagnosis of pemphigus.

No cases of intestinal tract infections or inflammatory bowel diseases were reported.

### Pharmacoepidemiology

#### A. Pemphigus Management

Briefly, regarding the drugs recommended by the latest guidelines for pemphigus management, it was found that no patient had received Dapsone or Cyclophosphamide as a steroid-sparing agent in the time period examined.

**Figure 3:**
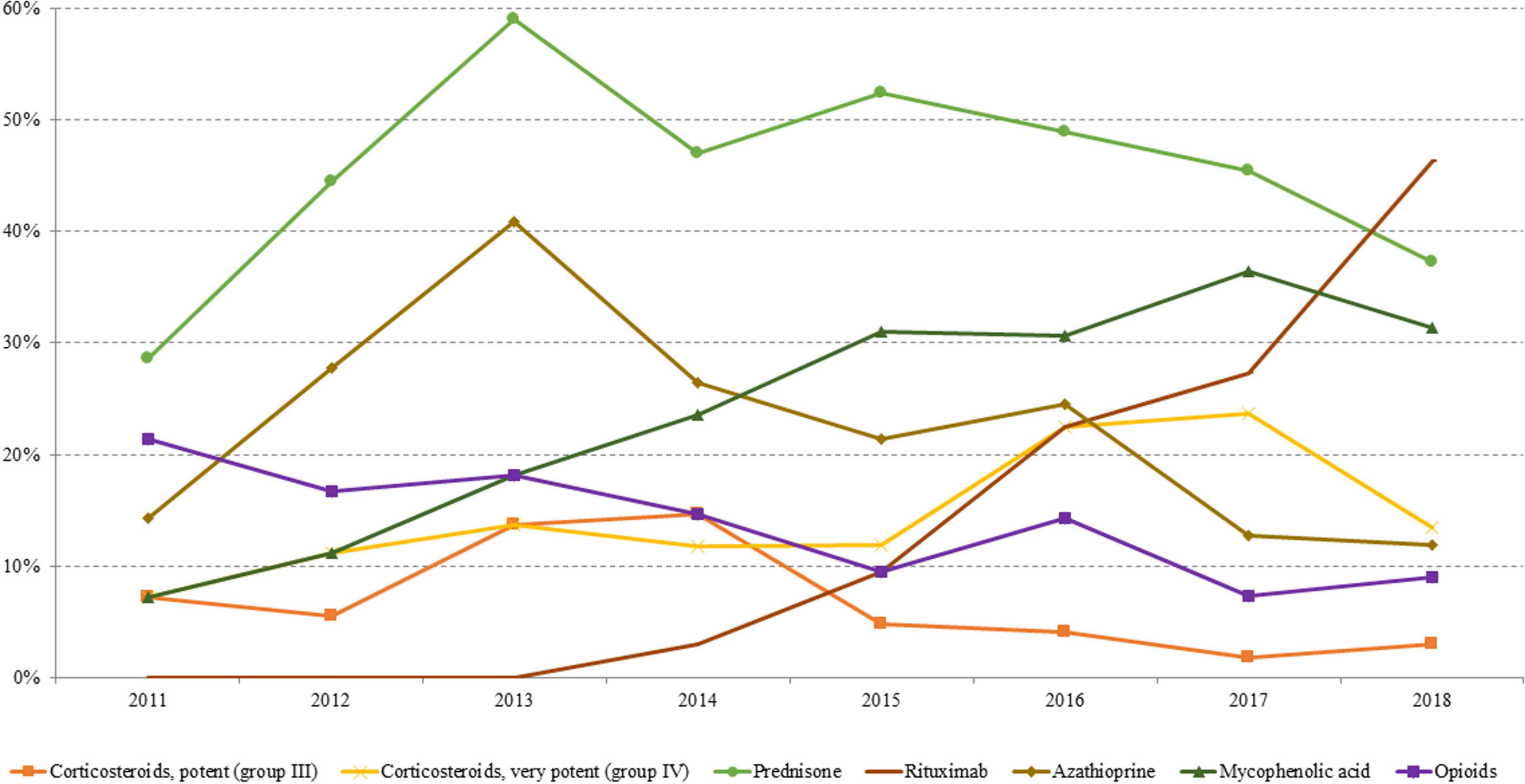
Prevalence trend (2011–2018) of the investigated drugs used for pemphigus management: topical corticosteroids, prednisone, azathioprine, mycophenolic acid, rituximab, and opioid.

The use of Mycophenolate Mofetil and Azathioprine as steroid-sparing therapies appeared to be almost stable throughout the years, albeit with a slight upward trend of Mycophenolate Mofetil compared to Azathioprine (**Figure 3**).

The use of these drugs did not suffer considerable changes following the introduction of RTX.

Specifically, in our study, RTX was started to be used from 2014, administered to one patient (2.9%) out of a total of 34 cases in study in that year.

Since 2015, there has been an increase in the use of RTX. At the end of the study, 46.3% of patients had received at least one cycle of RTX.

As expected, regarding the use of topical corticosteroids, a predilection in the use of high potency compared to medium potency was observed (in 2018, 13.4% and 3%, respectively).

About the use of opioids, recommended by the current guidelines for pain relief, we recorded a progressive decrease starting from 2011 (21.4%) until 2018 (9%) (**Figure 3**).

The intensity of use of Azathioprine and Mycophenolate, expressed as prescription/users (Pr/us; number of prescriptions of each drug/total number of cases with at least one dispensing per year), was also evaluated. Both drugs showed a steady trend between 2011 and 2018 ranging between 3.4 and 5.6 Pr/us and between 4.0 and 4.5 Pr/us for Azathioprine and Mycophenolate, respectively.

#### B. Therapies for the Management of Comorbidities in Patients With Pemphigus

PPIs and antibiotics were shown to be the most frequently consumed drugs among patients with pemphigus, with their use remaining substantially stable over the years (43.3% and 52.2% in 2018 for PPI and antibiotics, respectively).

Interestingly, the consumption of systemic antivirals started from 2016, whereas from 2011 to 2015, no patients were found to be prescribed with systemic antivirals. Interestingly, four of the eight cases that have at least one prescription of antivirals between 2016 and 2018 have received at least one prescription of RTX.

Since 2015, the prevalence of use of antifungal drugs for systemic use was about 2%.

**Figure 4:**
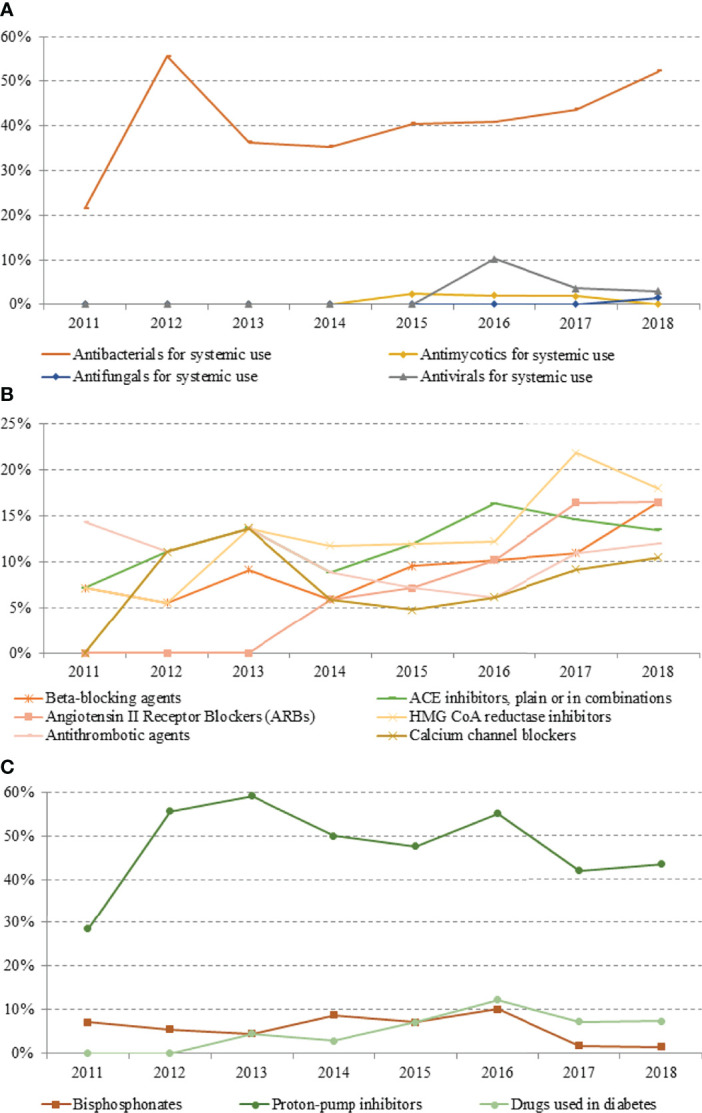
Prevalence trend (2011–2018) of therapies for the management of pemphigus-associated comorbidities: **(A)** antibacterial, antimycotics, antifungals and antivirals for systemic use; **(B)** beta-blockers, angiotensin II receptor blockers, ACE inhibitors, antithrombotic agents, HMG CoA reductase inhibitors, and calcium channel blockers; **(C)** bisphosphonates, proton-pomp inhibitors, and antidiabetic drugs.

Compared to PPI and antibiotics, there was a lower consumption (with a maximum of 12.2% in 2016) of insulin and non-insulin antidiabetic agents, anti-hypertensive drugs, such as beta blockers, calcium channel blockers, ACE inhibitors, and sartans (with a maximum of 16.4%, 11.1%, 16.3%, and 21.8%, respectively), and antithrombotic and anticoagulant agents (**Figure 4**).

## Discussion

The distribution of the different disease phenotypes within our cohort is in line with data reported in the literature (23), with a clear prevalence of PV over PF (4). Interestingly, the mean age at onset of pemphigus observed in our study was around 57, substantially overlapping to other epidemiological studies, such as one French series including 266 patients (24). Similar to that study, we did not observe substantial differences in terms of age at onset in relation to sex or pemphigus variant (24).

Differently, an English study including 138 patients with pemphigus demonstrated a significantly higher mean age at diagnosis (around 71 years) (25).

Pemphigus-specific mortality was estimated to be around 5% of patients in previous studies (3). In our series, 9% of the patients died during the study period. Death occurred in 10.2% of patients with PV, in 5.7% of patients with PF, and in one patient (corresponding to 33.3%) with PNP. Of note, the cause of death was available only for eight out of 14 patients, and in all these cases, it was not directly related to pemphigus itself. In another large series by Kridin et al., including 237 patients with pemphigus, with a slightly lower age at onset than our study, death was reported in 19.8% of patients with and 23.3% of patients with PF (19). The differences between these two studies might by due to the different sample sizes and/or to the different period of follow-up.

As expected, survival in our cohort was lower among patients with PV than PF. In addition, the survival rate decreased clearly with increasing age and was lower in male than female patients. Collectively, these data are similar to other previous studies in the literature (24).

As expected, there was a significant association between PV and anti-Dsg3 IgG antibodies as well as PF and anti-Dsg1 antibodies. Further, a significant drop in autoantibody titers between pemphigus diagnosis and the first 12 months of follow-up occurred in the vast majority of patients with PV (83.6% and 87.1% for anti-Dsg1 and anti-Dsg3, respectively) and PF (94.4% and 83.3% for anti-Dsg1 and anti-Dsg3, respectively). After 1 year of treatment, anti-Dsg1 antibodies were shown to decrease more in patients receiving RTX compared to those treated with other immunosuppressive agents. Because circulating antibodies reflect well the clinical activity and severity of the disease, our results confirm the efficacy of the various immunosuppressive treatments in pemphigus.

Concerning the treatment regimen, our study highlights the preferential use of Mycophenolate and Azathioprine as adjuvant steroid-sparing drugs for pemphigus management. Accordingly, none of the patients received treatment with other immunomodulatory drugs, such as dapsone, or immunosuppressive drugs, such as cyclophosphamide. Reasons include the fact that dapsone in not licensed for use in Italy; whereas cyclophosphamide showed an unfavorable safety profile compared to other steroid-sparing agents and has not shown meaningful superior efficacy compared to other drugs in randomized clinical trials. The increasing trend of RTX use observed in our sample is related to the accumulating evidence of efficacy of RTX in pemphigus (18, 26, 27), leading current guidelines to recommend the use of the drug as a first line option in pemphigus (5). Despite exciting results of RTX clinical trials (28), we observed that trend of prescriptions of Mycophenolate and Azathioprine remained substantially stable after its introduction in our cohort.

Among drugs used for the management of disease- or treatment-related comorbidities, PPI and antibiotics were the most frequently prescribed. The high consumption of PPI can be explained by the long-term use of corticosteroids in patients with pemphigus. In this regard, our series confirms the usefulness of this class of drugs in preventing severe and/or chronic gastrointestinal toxicity of systemic corticosteroids, as suggested by the low incidence of diseases of the upper gastrointestinal tracts.

Infections represent one of the most frequent comorbidities among patients with pemphigus and account for one of the main causes of pemphigus-related mortality (21, 29). The high consumption of antibiotics suggests that infections are also common in our patients’ cohort. Notably, it is arguable that most of the infections experienced by patients were of mild severity, as, except for two cases of pneumonia, both occurred in patients after pemphigus diagnosis, and there were no hospitalizations due to serious infections during the study period.

Notably, we recorded a very low consumption of antimycotic drugs, which suggests a low incidence of mycotic infections among patients. This is partly contrasting other studies in the literature, which demonstrated an increased susceptibility of patients with pemphigus to either dermatophyte or deep and systemic fungal infections (29).

Several studies reported herpes virus infections, including herpes zoster infections, as highly frequent in patients with pemphigus, especially in those receiving lymphocyte-targeting or B-cell depleting therapies, including Mycophenolate and RTX (30, 31). Moreover, herpes virus infections can also serve as a trigger for sudden worsening of pemphigus during immunosuppressive treatments (32, 33) and can be difficult to be recognized due to clinical similarities between herpes and pemphigus lesions (34). In our sample, despite a generally low consumption of systemic antivirals, half of the patients who were prescribed with this class of drugs had received treatment with RTX.

In our study, about 12% of patients with pemphigus received a diagnosis of malignancy; in about half of them, cancer occurred after pemphigus diagnosis. These data are congruent with other studies in the literature, suggesting a significant association between cancer and pemphigus, including non-paraneoplastic variants. The latter cases are referred to as malignancy-induced or malignancy-exacerbated pemphigus (20, 35–37). Collectively these findings highlight the importance of increased awareness about the potential risk of malignancy in patients with pemphigus. Symptoms, including weight loss, fatigue, and chronic fever, should raise suspicion and requires prompt recognition and appropriate diagnostic workup.

Although a strong systemic activation of coagulation has been mostly observed in bullous pemphigoid (38), similar to previous studies (21, 39, 40), we observed a higher incidence of cardiovascular and cerebrovascular diseases as well as thrombo-embolic events in our cohort, the vast majority of which reported after pemphigus diagnosis. Collectively, these findings suggest that patients with pemphigus may benefit from anti-hypertensive drugs, beta blockers, and anti-thrombotic drugs for preventing these potentially lethal adverse events.

Comorbid diseases including diabetes mellitus, cataracts, and glaucoma, observed in our patients, are presumably related to toxicity of long-term use of systemic corticosteroids.

Nutritional counseling may be thus important for patients to counterbalance the alterations of glucose metabolism due to systemic steroids.

Major strengths of the study include the large sample size and the monocentric design. The main limitation of the study relies on its retrospective design. As a result, long-term observation varies significantly among patients.

Other limitations include the fact that complete data on pemphigus-associated comorbidities and drug prescriptions were available for only 78 patients with pemphigus. For the same reason, we were not able to link the prescription database to the control population. A second limitation is that the regional prescription database does not collect data related to inpatient prescriptions. For this reason, for patients having experienced long hospitalization periods, the use of some drugs may have been underestimated. Finally, the causes of death were not available in the data exploited in this study and were retrieved only for some of the patients.

## Conclusions

This study represents the largest series of patients with pemphigus from a single referral center in Italy. This epidemiological study confirms that, despite pemphigus represents a prototype of organo-specific diseases, it frequently occurs in association with several comorbid diseases. Moreover, immunosuppressive treatments, patients often require additional treatments for managing these comorbidities. This finding highlights the importance of an integrated and multidisciplinary network for a correct management of patients and for optimizing costs.

Acute cerebro- and cardiovascular events and malignancies remain serious complications that dermatologists should keep in mind when managing patients with pemphigus.

## Data Availability Statement

The raw data supporting the conclusions of this article will be made available by the authors, without undue reservation.

## Ethics Statement

The studies involving human participants were reviewed and approved by Azienda USL Toscana Centro. Written informed consent to participate in this study was provided by the participants’ legal guardian/next of kin.

## Author Contributions

LQ, ACoi, RM, and MC contributed to conception and design of the study. LQ and ACoi organized the database. ACoi performed the statistical analysis. LQ, RM, and MC wrote the first draft of the manuscript. ACoi wrote sections of the manuscript. All authors contributed to manuscript revision, read, and approved the submitted version

## Funding

The Rare Diseases Registry of Tuscany is funded by the Tuscany Region, and it is managed by the Fondazione Toscana “Gabriele Monasterio” in collaboration with the Institute of Clinical Physiology of National Research Council.

## Conflict of Interest

The authors declare that the research was conducted in the absence of any commercial or financial relationships that could be construed as a potential conflict of interest.

## Publisher’s Note

All claims expressed in this article are solely those of the authors and do not necessarily represent those of their affiliated organizations, or those of the publisher, the editors and the reviewers. Any product that may be evaluated in this article, or claim that may be made by its manufacturer, is not guaranteed or endorsed by the publisher.
